# The Voluntary Hospitals' Opportunity

**Published:** 1912-11-30

**Authors:** 


### The Hospital

The Workers' Newspaper of Administrative Medicine and Institutional Life, Administration, National Insurance and Health.

No. 1378, Vol. LIII. SATURDAY, NOVEMBER 30, 1912. PRICE ONE PENNY.

### THE VOLUNTARY HOSPITALS' OPPORTUNITY.

One of the most difficult things in public life is to make an impending danger fully apprehended. Another is by disseminating knowledge, to bring the representatives of the interests threatened to the solution whereby it can be dispersed. We have felt it our duty as the upholders and defenders of the voluntary system of hospitals in this country—for The Hospital has for many years made articulate the thoughts of the most knowledgeable and experienced of their managers—to put forth our whole energies to place the business of these institutions, through the establishment of National Insurance, upon a financial basis which will be broad enough and strong enough, to enable them to undertake all the hospital treatment, which every class of persons resident in these islands, may require. We have proved by actual figures during the last twelve months that, for economical reasons which we have demonstrated but need not here repeat, the *pro rata* system, including loans to voluntary hospitals on a business basis, with adequate provision for defraying the cost of medical attendance, will uphold and strengthen the voluntary principle and render material service to the Government and the people of all classes. There can be no question that, neither the people who at present depend upon the voluntary hospitals for internal treatment when grievously ill, nor members of Parliament, quite apart from politics, nor the Government, can quietly stand inactive and wait until after January 16 next. They must now face and provide against the inevitable miseries and cruel sufferings, which will result to the workers throughout the country, from insured persons being deprived of the hospital benefit, without which neither the heads of families nor those dependent upon them can continue to maintain themselves in comfort and in health. This is not the place to repeat the grounds upon which we have urged the acceptance by the Government of the *pro rata* system, and its adoption by the managers of every British voluntary hospital of importance. A fortnight ago we urged hospital managers that the time for action had come, but it must be united action, and he "who seeks, and will not take when once 'tis offered, shall never find it more."

There is sound reason for the managers of the voluntary hospitals to be of good courage, for if they show a little statesmanship and patience now, whilst they unite in preparation for the adoption of the *pro rata* system, as advocated in the columns of The Hospital, we have reason to hope that the result must prove as remarkable, as it cannot fail to be acceptable, not only to the hospitals but to the whole population of these islands. Much surprise and some irritation have been caused by the evidence that the great voluntary hospitals of London, for example, have apparently gone to sleep and decided to let the present opportunity be lost through inaction. This impression has already, we understand, been attended by a separate step on the part of some of the larger of the smaller general hospitals ministering especially to the needs of the population within the outer boundaries of the metropolitan area. The managers of these hospitals have formed the impression that they cannot afford to accept the decision, as they understand it, of the larger hospitals to stand aside and do nothing. We should be in agreement with this view if this impression were justified by the facts. Happily, we are in a position to know that the larger hospitals have come to no such decision, and that there is a growing opinion amongst them that the *pro rata* system is one, with the safeguards it presents, that is worthy of the most favourable consideration on the part of the managers of all voluntary hospitals. This statement to-day is of the first importance, for the voluntary hospitals must stand together and work in unison, if the maximum of success is to be secured.

Now certain hospitals, who are not unnaturally impatient at the apparent delay, have called a meeting and decided to confer with the managers of the larger hospitals, so as to join hands and put the whole strength of the voluntary system behind this movement for a *pro rata* agreement in defence of the voluntary hospitals, and for the comfort of all who use the hospitals, by maintaining there, the best system of administration, from the patient's point of view, known to the world. Conversely we are glad to find that united action is favoured by the larger hospitals, and we hope that each of the large provincial and Scottish hospitals will immediately take steps through The Hospital, or by direct communication with the London Hospital, or one of the great hospitals of the metropolis, to join hands in support of the *pro rata* system. Unity is strength, and to combine without delay at this juncture means safety for the voluntary hospitals and enormous benefit to insured persons.

We have reason to know that, every individual member of Parliament has been made fully aware of the dangers which threaten the working classes of this country with ruinous and cruel miseries, until adequate hospital accommodation and benefit is secured for all insured persons. The press of all shades of opinion, too, have all the facts in their possession, and are fully seized with the knowledge of the emergency that has arisen, and of the paramount importance of finding and applying immediately the wise solution we have advocated. The fulness of knowledge of the actual position of these hospital questions to-day, which is possessed by Parliament and the members of the Government who are responsible for the policy to be pursued in this matter, makes it impossible to believe that, any one of these representative personages can shut his eyes to the plain facts of a situation, which the nation must face and resolve with wisdom and despatch.

Politicians of all parties are alike concerned in this business, and as we are told to repletion the present House of Commons is bored by the uninteresting character of the proceedings to which they are bound by the present procedure, they must welcome the opportunity which this great social question offers them to wake up the proceedings and prove that the present House of Commons can do the right thing promptly and irrespective of party when occasion arises. We have thought it might prove helpful to supply the facts of the present situation in a popular form under the title of "National Insurance and the Provision of Adequate Hospital Treatment for the Workers," which may be obtained at a nominal price through any bookseller or direct from The Scientific Press, Ltd., 28 Southampton Street, Strand, London, W.C.

